# Avoidance of urinary drainage during perioperative period of open elective colonic resection within enhanced recovery after surgery programme

**DOI:** 10.1093/gastro/goab006

**Published:** 2021-09-04

**Authors:** Yun Li, Zhi-Wei Jiang, Xin-Xin Liu, Hua-Feng Pan, Guan-Wen Gong, Cheng Zhang, Zheng-Rong Li

**Affiliations:** 1 Department of Gastrointestinal Surgery, The First Affiliated Hospital of Nanchang University, Nanchang, Jiangxi, P.R. China; 2 Jiangxi Institute of Digestive surgery, Nanchang, Jiangxi 330006, P.R. China; 3 Department of General Surgery, The Affiliated Hospital of Nanjing University of Chinese Medicine, Nanjing, Jiangsu, P.R. China

**Keywords:** colectomy, urinary catheterization, urinary retention, enhanced recovery after surgery

## Abstract

**Background:**

Urinary catheterization (UC) is a conventional perioperative measure for major abdominal operation. Optimization of perioperative catheter management is an essential component of the enhanced recovery after surgery (ERAS) programme. We aimed to investigate the risk factors of urinary retention (UR) after open colonic resection within the ERAS protocol and to assess the feasibility of avoiding urinary drainage during the perioperative period.

**Methods:**

A total of 110 colonic-cancer patients undergoing open elective colonic resection between July 2014 and May 2018 were enrolled in this study. All patients were treated within our ERAS protocol during the perioperative period. Data on patients’ demographics, clinicopathologic characteristics, and perioperative outcomes were collected and analysed retrospectively.

**Results:**

Sixty-eight patients (61.8%) underwent surgery without any perioperative UC. Thirty patients (27.3%) received indwelling UC during the surgical procedure. Twelve (10.9%) cases developed UR after surgery necessitating UC. Although patients with intraoperative UC had a lower incidence of post-operative UR [0% (0/30) vs 15% (12/80), *P *=* *0.034], intraoperative UC was not testified as an independent protective factor in multivariate logistic analysis. The history of prostatic diseases and the body mass index were strongly associated with post-operative UR. Six patients were diagnosed with post-operative urinary-tract infection, among whom two had intraoperative UC and four were complicated with post-operative UR requiring UC.

**Conclusion:**

Avoidance of urinary drainage for open elective colonic resection is feasible with the implementation of the ERAS programme as the required precondition. Obesity and a history of prostatic diseases are significant predictors of post-operative UR.

## Introduction

The enhanced recovery after surgery (ERAS) strategy is the earliest and most widely applied in colorectal surgery, the central purpose of which is to decrease stress and accelerate recovery via several perioperative interventions that preserve physiological function [1]. Optimal management of various catheters, which comprise a nasogastric tube, a drainage tube of the peritoneal cavity, and urinary drainage, is essential in the ERAS system. For a long time, urinary catheter placement has been a standard and conventional practice for colorectal operations, especially in the presence of traditional analgesia. Urinary catheterization (UC) functions in bladder decompression, urine-output measurement, and urinary-retention (UR) treatment [2]. However, with the wide acceptance of the ERAS concept by many surgeons in recent years, the disadvantages of urinary drainage during perioperative care have gained increasing attention.

The incidence of post-operative urinary-tract infection (UTI) is strongly associated with the duration of an indwelling UC in placement [3–5]. The incidence of bacteriuria 24 hours after UC is ∼5%–10%, with morbidity increasing while the duration of urinary drainage is prolonged [6]. Furthermore, the presence of a UC could affect the implementation of other ERAS-recommended procedures, such as early mobilization, limited intravenous-fluid treatment, and multimodal control of patient discomfort. Hence, several studies have explored the possibility of avoidance or early removal of UC to minimize its influence on post-operative recovery [7–9]. Laparoscopic colonic resection without indwelling UC is safe and feasible [7]. Even for rectal surgery, removal of UC on the first post-operative day is realizable [9].

At present, surgeons are inclined to select open colonic resection for some cases with large advanced tumours, especially those accompanied by obstruction, in order to facilitate digestive-tract reconstruction and check the sufficiency of the surgical margin [10–12]. In theory, abdominal open operations that are associated with invasiveness, stress, and strong analgesia may be at higher risk of post-operative UR in contrast to the laparoscopic approach. Consequently, a hypothesis that UC may be avoided in perioperative management by applying the ERAS protocol for open elective colonic surgeries can be framed. However, the relevant research on perioperative urinary drainage within the ERAS programme for colonic open operations is lacking. The current study aims to summarize our experience of urinary-drainage management employed in the ERAS protocol for open elective colonic resection, focusing on the feasibility of avoiding UC and the risk factors of post-operative UR.

## Patients and methods

### Study design and subjects

This retrospective case-series study enrolled 110 patients with colonic cancer who underwent elective open radical operation in the First Affiliated Hospital of Nanchang University and the Affiliated Hospital of Nanjing University of Chinese Medicine between July 2014 and May 2018. This study was approved by the institution review board (IRB) of Jinling Hospital (IRB number: 2017NZKY-012–02). Informed consent was obtained from each patient before the planned treatment. Consent to report individual patient data was also obtained from the participants. The exclusion criteria for this study were as follows: (i) patients >85 or <18 years old; (ii) patients undergoing laparoscopic or robot-assisted operation; (iii) patients undergoing colonic resection combined with operation in other organs; (iv) patients receiving UC or suffering from UTI prior to the operation; (v) emergency surgery; (vi) patients not enrolled into the ERAS clinical pathway owing to not acquiring informed consent.

### ERAS protocol

The ERAS protocol employed in this study was performed in compliance with the universal guidelines of ERAS practice [1, 13] and the key components were presented in brief as follows: oral intake of carbohydrate prior to operation, avoidance of bowel preparation and nasogastric tube, post-operative early ambulation, early oral nutritional intake after surgery, limited intravenous-fluid treatment, and multimodal analgesia.

On the operation day, all patients were asked to void before entry into the operating room. Intraoperative UC was performed in the case of a complicated surgical procedure with an anticipated duration of >180 minutes or upon the request of an anaesthetist for intensive fluid treatment. Among these patients, the indwelling catheter was removed at the end of operation before transferring to the resuscitation unit. The amount of intraoperative fluids infused was limited to 6–8 mL/kg/h. The epidural and intravenous patient-controlled analgesia pumps were abolished and the multimodal analgesia mentioned above without strong opioids was applied instead.

During the post-operative recovery, the amount of intravenous fluids was controlled at between 1,000 and 1,500 mL on the first day after surgery and ≤1,000 mL per day afterwards. Pain intensity was evaluated at 24 hours after surgery by using the patients’ self-report of the Numeric Rating Scale, by which the pain intensity was rated by patients with a visual assistance on a scale from 0 to 10. Patients reporting pain scores of 0–3 were identified as ‘no pain or mild pain’, whereas 4–10 indicated ‘moderate or greater pain’ [14].

Micturition was monitored carefully by our nursing team. Bedside bladder sonography was performed in case of patients’ complaint of difficulty in urinating. In the event of post-operative UR, a transurethral catheter was inserted. During the UC, the tube was maintained in occlusion with once open every 2 or 3 hours. Meanwhile, urinalyses were performed to monitor the occurrence of UTI. When spontaneous micturition was achieved, the indwelling catheter was removed immediately.

### Outcome measurement

The primary end points of this study were post-operative UR, defined as inability of spontaneous micturition and a dilated bladder scan of >400 mL by sonography, and its risk factors. The incidence of UTI, which was diagnosed in line with the ACS NSQIP standardized definition of post-operative UTI (Table 1) [15], was the secondary outcome.

**Table 1.** Standardized definition of post-operative urinary-tract infection in ACS NSQIP [15]

Criterion 1	Criterion 2
Either one of the following items:	Two of the following items:
Fever (>38°C)	Fever (>38°C)
Urgency	Urgency
Frequency	Frequency
Dysuria	Dysuria
Suprapubic tenderness	Suprapubic tenderness
And a urine culture of >100,000 colonies/mL urine with ≤2 species of organisms	And any of the following items:
	Dipstick test result positive for leukocyte esterase or nitrate
	Pyuria (>10 WBCs/mm^3^ or >3 WBCs/high-power field of unspun urine)
	Organism seen on Gram stain of unspun urine
	2 urine cultures with repeated isolation of the same uropathogen with >100 colonies/mL urine in a nonvoided specimen
	Urine culture with >100,000 colonies/mL urine of a single uropathogen in a patient being treated with appropriate antimicrobial drugs
	Physician’s diagnosis of UTI

Post-operative urinary-tract infection must meet either of the two criteria above.

### Statistical analysis

Final data were expressed as mean ± standard (SD) or number (percentage). LSD *t*-test was applied for comparisons of continuous variables. The Chi-square test or Fisher exact test was used to compare categorical variables. Logistic regression was involved to identify risk factors for UR after open colonic resection. Differences at *P* < 0.05 were considered statistically significance.

## Results

### Demographics and clinicopathologic characteristics

A total of 110 patients were enrolled in the current study. There were 61 males and 49 females with a mean age of 59.1 ± 12.3 years. The tumour staging was in accordance with the 8th Edition Cancer Staging System presented by the Union for International Cancer Control, with 13 cases (11.8%) in stage I, 55 (50%) in stage II, 38 (34.5%) in stage III, and 4 (3.6%) in stage IV. Twenty-four patients (21.8%) had undergone neoadjuvant chemotherapy (CapeOx regimen: oxaliplatin plus capecitabine) prior to operations. Health education on the ERAS programme was performed preoperatively for all patients, who also signed the informed consent voluntarily. The surgical procedures comprised right hemicolectomy in 57 cases (51.8%), transverse colectomy in 4 cases (3.6%), left hemicolectomy in 14 cases (12.7%), and sigmoid colectomy in 35 cases (31.8%). The reconstructions of the digestive tract were performed by using circular staplers. The average operative time was 166.0 ± 34.2 minutes (Table 2).

**Table 2.** Demographic, clinicopathologic, and perioperative characteristics of this cohort of patients with colon cancer

Characteristic	Value (*n* = 110)
Male sex, *n* (%)	61 (55.5)
Age, years, mean ± SD	59.1 ± 12.3
Body mass index, kg/m^2^, mean ± SD	23.3 ± 3.2
ASA classification, *n* (%)	
I	12 (10.9)
II	98 (89.1)
Operation time, minutes	166.0 ± 34.2
Intraoperative fluid administration, mL, mean ± SD	1,284.5 ± 356.6
Intraoperative urine volume, mL, mean ± SD^a^	185.7 ± 130.1
Type of operation, *n* (%)	
Right hemicolectomy	57 (51.8)
Transverse colectomy	4 (3.6)
Left hemicolectomy	14 (12.7)
Sigmoid colectomy	35 (31.8)
Tumour stage, *n* (%)	
I	13 (11.8)
II	55 (50.0)
III	38 (34.5)
IV	4 (3.6)
Indwelling UC during perioperative period, *n* (%)	
Intraoperative UC	30 (27.3)
Post-operative urinary retention necessitating UC	12 (10.9)
Post-operative complications, *n* (%)	
Urinary-tract infection	6 (5.5)
Pulmonary infection	3 (2.7)
Anastomotic leakage/intra-abdominal infection	1 (0.9)
Mild anastomotic bleeding	2 (1.8)

SD, standard deviation; ASA, American Society of Anesthesiologists; UC, urinary catheterization.

a Data from 30 patients undergoing intraoperative UC.

### Perioperative outcomes

All patients enrolled followed the ERAS protocol and no one quitted the programme midway. Sixty-eight patients (61.8%) underwent surgery without any perioperative UC. Thirty patients (27.3%) received indwelling UC intraoperatively because of prolonged operative time or need for higher intravenous-fluid resuscitation and haemodynamic monitoring. All intraoperative UCs were terminated at the end of operations. Twelve (10.9%) cases developed post-operative UR after a median of 15 (range 2–40) hours, among which the mean volume of bladder urine detected by sonography at the time of UC was 462.5 (range 400–600) mL (Table 2). All patients with post-operative UR underwent indwelling UC and had successful micturition within 72 hours after catheterization. The mean duration of urinary drainage was 29.6 (range 10–65) hours.

Six patients were diagnosed with UTI, among whom two had intraoperative UC and four had post-operative UR necessitating UC. The patients without UC, regardless of whether intraoperative or post-operative, did not experience UTI. Besides UTI, post-operative complications included pulmonary infection (*n* = 3), subsequent intra-abdominal infection caused by anastomotic leakage (*n* = 1), and mild anastomotic bleeding (*n* = 2). All these operative complications were cured non-surgically. As for the pain assessments at 24 hours post-operatively, 99 patients experienced ‘No pain or mild pain’, while 11 patients experienced ‘Moderate or greater pain’.

### Factors associated with post-operative UR

Univariate analysis showed that high body mass index (BMI), a history of prostatic diseases including benign hyperplasia and inflammation, and intraoperative UC were associated with post-operative UR after open colonic resection (Table 3). Nevertheless, multivariate analysis revealed that only the history of prostatic diseases and high BMI, not intraoperative UC, were independent risk factors for post-operative UR (Table 4).

**Table 3.** Univariate analysis for risk factors for urinary retention after open colonic resection

Characteristic	Post-operative urinary retention		*P*-value
	Yes (*n* = 12)	No (*n* = 98)	
Gender, *n* (%)			0.543
Male	8 (66.7)	53 (54.1)	
Female	4 (33.3)	45 (45.9)	
Age, years, mean ± SD	62.8 ± 14.7	58.7 ± 12.0	0.374
Body mass index, kg/m^2^, mean ± SD	25.7 ± 3.1	22.9 ± 3.1	0.011
History of prostatic diseases, *n* (%)	3 (25.0)	1 (1.0)	0.004
ASA classification, *n* (%)			0.618
I	2 (16.7)	10 (10.2)	
II	10 (83.3)	88 (89.8)	
Operative time, minutes, mean ± SD	177.1 ± 44.3	164.6 ± 32.6	0.363
Intraoperative fluid administration, mL, mean ± SD	1,183.3 ± 442.8	1,296.9 ± 345.4	0.407
Intraoperative urinary catheterization, *n* (%)			0.034
Yes	0 (0)	30 (30.6)	
No	12 (100)	68 (69.4)	
Pain intensity, *n* (%)			0.185
No pain or mild pain	9 (75.0)	90 (91.8)	
Moderate or greater pain	3 (25.0)	8 (8.2)	
Types of operation, *n* (%)			0.811
Right hemicolectomy	7(58.3)	50 (51.0)	
Transverse colectomy	0 (0)	4 (4.1)	
Left hemicolectomy	2 (16.7)	12 (12.2)	
Sigmoid colectomy	3 (25.0)	32 (32.7)	
Tumour stage, *n* (%)			0.811
I	1 (8.3)	12 (12.2)	
II	6 (50.0)	49 (50.0)	
III	4 (33.3)	34 (34.7)	
IV	1 (8.3)	3 (3.1)	

SD, standard deviation; ASA, American Society of Anesthesiologists.

**Table 4.** Multivariate-regression analysis for risk factors of urinary retention after open colonic resection

Variable	B	S.E.	Wald	df	*P*	Odds ratio (95.0% CI)
Body mass index	0.482	0.181	7.048	1	0.008	1.619 (1.134–2.31)
Prostate medical history	4.537	2.055	4.876	1	0.027	93.44 (1.665–5.243E3)
Intraoperative urinary catheterization	–18.713	6,539.372	0.000	1	0.998	0.000

## Discussion

At present, UC is the most commonly used approach to prevent post-operative UR after major abdominal surgery, which was reported to have an incidence rate ranging from 3.8% to 38% [16–20]. Although early UC removal on the first post-operative day is feasible and safe for patients without high risk factors for UR [2], the possibility of avoidance of UC must still be observed during the perioperative period within the ERAS protocol, one of the main components of which is optimal management of various catheters. Meanwhile, a novel viewpoint that UC removal at the end of colonic operation may not increase the risk of UR has been supported by some scholars [16]. Hence, in the current study, we investigated the potential predictors of UR after open colonic resection within the ERAS protocol and explored a modified clinical management of UC, in which perioperative UC was abolished or for some special patients removed immediately when the operation was finished.

The underlying pathogenesis for post-operative UR in patients undergoing colonic resection is multi-factorial and complicated, including primary disease of patient, anaesthesia, operation, post-operative analgesia, and perioperative fluid treatment. Due to more invasiveness and discomfort following open surgery as compared to laparoscopic surgery, the emphatic use of analgesia, especially strong opioid drugs and epidural analgesia, may increase the incidence rate of post-operative UR. Although some studies have explored the feasibility and safety of abandonment or early removal of UC in patients undergoing laparoscopic colorectal surgery [7, 9], few surgeons have attempted to reduce UC use for patients undergoing open colonic operations, which are associated with stronger post-operative analgesia and a higher incidence of post-operative UR in contrast to the laparoscopic approach. Our experience summarized in this research supports the feasibility of perioperative management without UC for open colonic surgery, which is regarded as a beneficial measure for post-operative rehabilitation and an important improvement of the ERAS programme.

Another remarkable finding of the current study is that the incidence rate of UR after colonic surgery within our ERAS pathway was only 10.9%, which was much lower than the data reported in literature concerning colorectal surgery without perioperative ERAS management, in which the overall incidence of post-operative UR ranged from 22.4% to 33.3% [17, 18]. With respect to perioperative ERAS management, the incidence rate of UR after colonic surgery ranges from 14% to 18% [19]. This favourable result has two explanations. First, multimodal analgesia was applied in our ERAS programme in the absence of epidural analgesia, which was usually advocated by most ERAS guidelines and was considered as a significant predictor for post-operative UR. Previous research also verified that early removal of UC in the presence of thoracic epidural analgesia can significantly elevate the risk of UR after colorectal surgery [20]. Second, limited intravenous-fluid treatment was carried out strictly in the current cohort. The overall rate of UR after surgery, regardless of abdominal or pelvic operations, is associated with increased perioperative fluid administration [19]. A high volume of fluid treatment could also lead to the overdistension of the bladder wall and the inability of detrusor contraction [21]. Thus, the American Society of Colon and Rectal Surgeons recommends that perioperative fluid administration be restricted to reduce UR incidence after ambulatory anorectal surgery [22]. However, the volumes of intraoperative fluid administration in the current study showed no significant link with the occurrence of UR, which may result from the universal application of limited fluid treatment for all the patients in this research. A control group receiving traditional fluid treatment will be required in further investigation to determine the relationship between fluid volume and post-operative UR.

In the present cohort, bladder overdistension exceeding 400 mL as detected by sonography was considered as an important indicator for necessitating UC because the incidence of persistent bladder dysfunction increases significantly with bladder volume >500 mL for a period [23], among which bladder ischaemia and decreased detrusor contractility were thought to be the main mechanisms [24]. In the present study, obesity and a history of prostatic diseases were significant predictors for UR after open colonic resection within the ERAS protocol. Some mechanisms may be involved in the relationship between the predictive factors and post-operative UR. First, high stress on the pelvic floor and serious spasm of the urethral sphincter after surgery are common in obese patients and those with a history of prostatic diseases. Second, patients with prostatic hyperplasia following major operations are susceptible to urethral obstruction and neuromuscular dysfunction of the urinary tract [25]. These findings suggest that vigilance should be paid to patients with the two risk factors during the perioperative period to detect UR early on and intervene as soon as possible.

Post-operative bacteriuria is strongly associated with the intravesical placement of the indwelling catheter. Previous reports estimated that ∼67%–80% of hospital-acquired UTIs are related to the indwelling urinary catheter [26–28]. A long duration of catheterization indicates a high morbidity of bacteriuria [29]. In the present study, all the post-operative UTIs were observed in patients with UC. This result demonstrates that a decreased rate of perioperative UC might be beneficial for controlling hospital-acquired UTI.

This study has some limitations that should be strengthened in further investigations. First, this study has a retrospective and observational design without a strict control cohort. Invalid association resulting from selection bias may account for the opposite significance of intraoperative UC revealed by univariate and multivariate analyses. All patients receiving intraoperative UC were without history of prostatic diseases, which was proved to be a strong risk factor for post-operative UR in this study. A prospective randomized trial employing a larger cohort should be planned and carried out strictly in the future to obtain objective results and provide strong evidence. Second, although all the cases enrolled in the current research were treated strictly within the ERAS pathway; differences in the patients’ compliance to the ERAS protocol, even if they were mild, were ignored. Thus, a stratification analysis is required to determine the impact of patients’ obedience to the ERAS protocol on the incidence rates of post-operative UR and UTI.

In conclusion, urinary drainage can be avoided during the perioperative period for open colonic surgery by using the ERAS protocol including multimodal analgesia in the absence of epidural analgesia and limited intravenous-fluid treatment. Patients with obesity or a history of prostatic diseases are predisposed to a high risk of post-operative UR.

## Authors’ Contributions

Z.R.L. planned the study. Z.R.L. and Y.L. conducted the study. Y.L. and X.X.L. collected and analysed the data and drafted the manuscript. H.F.P., G.W.G., and C.Z. helped in collecting the data. Z.W.J. supervised the study and revised the manuscript. All authors read and approved the final manuscript.

## Funding

This work was supported by the China Postdoctoral Science Foundation [grant number 2018M633694], National Natural Science Foundation of China [grant number 81500417], Social Development Fund of Jiangsu Province [grant number BE2015687], and Jiangsu Planned Projects for Postdoctoral Research Funds [grant number 1701159C].
